# 

**Published:** 1893-11-25

**Authors:** 


**Notice to Advertisers and Subscribers.**

All Advertisements, Orders for Copies of THE HOSPITAL, and Business Communications should be addressed to The Publisher, *not to the Editor*, THE HOSPITAL, 428, Strand, W.C.

The Cost of Subscription, post free, is as follows:—Per week, 2½d.; per quarter, 2s. 6d.; per half-year, 5s.; per annum, 8s. 6d. Foreign:—Per Annum, 10s. 8d.; payable, in all cases, in advance.

**OUR NEW OFFICES,**

428, Strand, W.C., will henceforth be the Office of this Journal.

**Notices of Births, Deaths, and Marriages** are inserted in this column at a charge of 2s. 6d. for an announcement not exceeding four lines.

### NOTICE TO CORRESPONDENTS.

All MS., Letters, Books for Review, and other matters intended for the Editor should be addressed THE EDITOR, THE LODGE, PORCHESTER SQUARE, LONDON, W.

The Editor cannot undertake to return rejected MS., even when accompanied by stamped directed envelope.

**"Burdett's Hospital Annual," 1893.**

Fourth year of publication. 700 pp. Boards, gilt lettering. A most complete guide to British, Colonial, Indian, and American Hospitals, Dispensaries, Nursing and Convalescent Institutions, and Asylums. Price 5s. Published by THE SCIENTIFIC PRESS (Limited), 428, Strand, W.C.

**NOTICE.—The Index for Vol. XIV. (ending Sept. 30th, 1893) is on sale at the Office of this Journal, price 2½d., post free.**

### SCRAPS AND GLEANINGS.

THE Children's Hospital, Temple Street, Dublin, this year attained its majority.

THE number of patients at the Royal Ear Hospital, Frith Street, during October, were 824.

A STATE museum is being erected at Pretoria, chiefly with the view to obtaining a collection of the Transvaal fauna.

BRUSSELS UNIVERSITY will shortly have a Laboratory of Psychological Physics, endowed by private munificence.

A CARNIVAL in aid of the funds of the Cardiff Infirmary has been arranged to take place early in the present month.

A DONATION of £2,000 has been received by the Newcastle Infirmary authorities out of the estate of the late Lady Armstrong.

A CONCERT was lately given at the Durham Town Hall in order to benefit the funds of the Friendly Societies Convalescent Home at Grange-over-sands.

THE Bangor Out-Patient Infirmary is now closed for the reception of extern cases, and in place of it the Provident Dispensary in Plas.lynd Terrace is opened.

A PUBLIC meeting was called recently at the Royal Institution, Swansea, for the purpose of proposing a convalescent home for Swansea and the surrounding district.

A MOVEMENT is on foot for holding a hospital exhibition next year in Woodhouse Park, Uxbridge Road. Major Wyndham Malet being the organizer of the scheme.

AT the latest meeting of the Governors and Guardians of the Rotunda Hospital at Dublin, the bank-book, which was laid before the meeting showed an overdraft of £1,300.

THE annual dinner of the staffs of the Richmond, Whitworth, and Hardwicke Hospitals took place recently in Dublin, the Right Hon. Joseph Meade occupying the chair.

A HANDSOME set of silver table candlesticks, with central candelabrum, has been presented to the Treasurer of the Royal Victoria Hospital, Bournemouth, upon his retiring from office.

A FREE bed has been presented to the Liverpool Hahnemann Hospital, through a public collection, as a memorial to the late Dr. Drydale. This eminent scientist was the originator of homœopathic dispensaries in Liverpool.

THE Linden Convalescent Home at Blackrock, which has been closed during the autumn months, is reopened now for the reception of patients. This institution is a valuable auxiliary to the St. Vincent's Hospital, Dublin.

THE balance-sheet for 1892 of the South Molton Sick Nurse Association showed an outstanding liability of £2 9s. It was resolved at the annual meeting of this association that this debt should be paid out of this year's subscriptions.

THE fifth annual ball in aid of the funds of the Royal Free Hospital, Gray's Inn Road, will take place at the Holborn Town Hall on Thursday, November 30th, under the patronage of the Duke of Portland, the Duke of Fife, &c.

AT the annual meeting of the Edinburgh Royal Infirmary Samaritan Society, it was stated that the subscriptions had remained stationary, and the Committee are consequently greatly hampered by the smallness of the means at their disposal.

THE work at the Royal Infirmary at Liverpool has extended so much during recent years, that the cost of maintenance has necessarily been increased also. A special appeal for further subscriptions is being urged upon the Liverpool citizens.

THE late Professor Olivieri, of Naples, left a very large estate, which he had acquired from his practice. By his will he made several munificent bequests, the Naples Blind Asylum and the Pellegrini Hospital benefiting considerably through this means.

THE directors' report of the Longton Cottage Hospital states that, notwithstanding all the difficulties which the working classes have had to contend with, the financial result, as far as the hospital is concerned, is more satisfactory than they had anticipated.

THE authorities of the Dundee Eye Institution propose that an arrangement should be consolidated with the General Infirmary of the town, through which accommodation could be provided in the wards for any serious surgical cases occurring in the former institution.

THE Sanitary Committee of Lynn has received a deputation from the Poor-Law Guardians calling attention to the necessity of erecting an infectious diseases hospital, The Borough Surveyor has been instructed forthwith to prepare plans for a suitable iron building for this purpose.

AT a meeting of the Joint Hospital Board, held at Margate, it was agreed upon to purchase a site for the new hospital. It is to be expected opposition will be raised to this particular piece of land being chosen, the price asked for the six acres being £1,500, and it is close to the present hospital.

THE Scottish Association for the Medical Education of Women has collected the sum of £50, to be presented to the Royal Infirmary, Edinburgh, in recognition of the fact that the wards of the infirmary are now thrown open to women students who wish to qualify in clinical instruction.

AT the annual meeting of the Brackley Cottage Hospital it was stated that 48 patients had been treated during the past year. The secretary, in his report of the general condition of the hospital, claims that in comparison with other institutions of their kind it was managed on a most economical system.

THE West of England Eye Infirmary, at Exeter, held its eighty-fifth annual meeting recently, the report of which, during the last twelve months, shows that the financial position had been fairly well sustained. The ordinary income, while differing in some of the items in the aggregate was within £8 of the amount received in 1891-2.

THE renewed attempt of the Shirley Freemantle Local Board to obtain the use of the hospital erected at Westend for their cases of infectious disease has failed. The idea is now mooted of establishing a permanent hospital for this purpose, in which the joint districts of Shirley, South Stoneham, and Eastleigh would amalgamate for its support.

### PUBLISHERS' ANNOUNCEMENTS.

### THE NURSES' CASE BOOK.

**FOR USE IN HOSPITAL AS WELL AS PRIVATE NURSING.**

Demy 16mo (for the apron pocket), 72pp., strong boards. Second Edition. Price 6d., or 4s. 6d. per dozen (post free).

**SPECIMEN PAGE FILLED IN.**

A Book of Tables specially prepared for use by Nurses in the Ward and in the Sick-Room. Containing Twenty-Six complete Tables, each being ruled to last one week, for keeping an exact record of a Patient's condition, including notes on Temperature, Pulse, Respiration, Motions, Age, Sex, Name, Address, Occupation, Treatment, History, Name of Medical Attendant, Number of Case, and Date of Admission.

Nurses should combine together and order parcels of over a dozen to profit by the special discount terms—*i.e.*, 4s. 6d. per dozen (post free).

**London: THE SCIENTIFIC PRESS, Ltd., 428, STRAND, W.C.**

### BOOKS FOR INSTITUTIONS.

**Ophthalmic Nursing.** An important new work on a new subject, profusely Illustrated. By Sydney Stephenson, M.B., F.R.C.S.E. With list of instruments required, glossary, &c. Crown 8vo, 200 pp. Price 3s. 6d.

**Surgical Ward-Work and Nursing.** A Practical Manual of Clinical Instruction for Nurses and Students. By Alexander Miles, M.D. (Edin.), C.M., F.R.C.S.E. Over one hundred illustrations. Demy 8vo, 200 pp. Price 3s. 6d.

**Hospital Sisters and their Duties.** By Eva C. E. Lückes. 2nd Edition. 2s. 6d.

**The Theory and Practice of Nursing.** By Percy G. Lewis, M.D. 4th Edition. 3s. 6d.

**Mental Nursing.** By William Harding, M.B. 2s. 6d.

**Outlines of Insanity.** By Francis H. Walmsley, M.D. Popular Edition. 1s. 6d.

**Art of Feeding the Invalid.** By a Medical Practitioner and a Lady Professor of Cookery. 3s. 6d.

**Art of Massage.** By A. Creighton Hale. 70 illustrations. 6s.

**The Nurse's Dictionary of Medical Terms and Nursing Treatment.** By Honnor Morten. 10th thousand in preparation.

**Hospitals and Asylums of the World.** By Henry C. Burdett, in four volumes and a portfolio of plans, £8 8s.

**Burdett's Hospital Annual,** and Year-book of Philanthropy, 1893. By Henry C. Burdett. 5s.

**Accounts, Audit and Tenders,** for Hospitals and Institutions, The Uniform System of. By Henry C. Burdett. 6s.

**Account Books for Institutions.** Series No. 1: Complete set of Eight Books, price £10. Series No. 2: Set of Four Books (for small Institutions), price £4. (Full catalogue and particulars post free).

**Surgical Ward-Work and Nursing.** By Alexander Miles, M.D. (Edin.), C.M., F.R.C S.E. (In the Press.)

**Suffering London:** A Book about Hospitals and their Needs. By A. Egmont Hake, with an Introduction by Walter Besant. 3s. 6d.

**Ministering Women:** The Story of the Royal National Pension Fund for Nurses. By George W. Potter, M.D. 2s. 6d.

**Cottage Hospitals:** General, Fever, and Convalescent. By Henry C. Burdett. (3rd Edition in the Press.)

London: THE SCIENTIFIC PRESS (Limited), 428, Strand, W.C.

### PUBLISHERS' ANNOUNCEMENTS.

READY THIS DAY.

IMPORTANT NEW TEXT BOOK OF OPHTHALMIC NURSING.

Crown 8vo. Cloth Gilt, pp. 200, profusely illustrated, and with many woodcuts. Price 3s. 6d., post free.

### OPHTHALMIC NURSING.

BY SYDNEY STEPHENSON, M.B., F.R C.S.E., Surgeon to the Ophthalmic School, Hanwell, W., &c., &c.

As yet no book has been published dealing with nursing in diseases of the eye.

As a text book, "Ophthalmic Nursing" will be invaluable to Nurses desiring to qualify in this particular branch of nursing.

The managers of ophthalmic institutions are invited to adopt this work as a text-book for their nursing staffs.

The book contains a frontispiece, "Vertical Section of the Eye-ball and Eye lids," and sixty-one other illustrations.

It is concisely yet exhaustively written, and its method of treatment and style will render it especially serviceable to the Nursing Profession.

READY THIS DAY.

Demy 8vo., 200 pp.; Cloth Gilt, with 199 Illustrations. Price 3s. 6d., post free.

### SURGICAL WARD WORK AND NURSING.

By ALEXANDER MILES, M.D. (Edin.), C.M., F.R.C.S.E.

A Manual of Clinical Instruction for Nurses and Students.

Profusely Illustrated with One Hundred and Ninety-Nine Illustrations. This will be of the greatest service as a text-book of Surgical Ward Work and Nursing.

It deals more minutely and in more detail than any other known work on the same subject.

As a text-book it is well suited to the requirements of Nurses in Accident Wards; and it contains much information that has been considered too elementary to include in most similar treatises.

Orders may be sent through any bookseller; or copies will be dispatched by return of post on receipt of the published price (3s. 6d.)

**London: The Scientific Press (Limited), 428, Strand, W.C.**

### Medical Vacancies and Appointments.

### APPOINTMENTS.

A. Ashby, M.B.Lond., F.R.C.S.Eng., reappointed Medical Officer of Health to the Reading Town Council. Dr. Bannatyne, appointed Parochial Medical Officer for Southend, vice William Seright, M.B., C.M.Glasg., resigned. Mr. C. E. Bashall, appointed Medical Officer for the Eighth and Ninth Districts of the Kingsbridge Union. J. Guthrie Blandford, M.R.C.S., L.R.C.P., appointed Resident Medical Officer to the West End Hospital for Diseases of the Nervous System, Paralysis, and Epilepsy, Welbeck Street. L. Buggy, L.R.C.P.I. and L.M., L.R.C.S.I., appointed Assistant Medical Officer to the District Asylum, Kilkenny. Ann Elizabeth Clark, M.D.Berne, L.R.C.P.I., appointed an Acting Physician to the Children's Hospital, Birmingham. H. G. O. Collett, L.R.C.P.Lond., M.R.C.S.Eng., appointed Medical Officer for the Marske District of the Guisborough Union. D. S. Dunn, B.A., M.D., M.Ch., appointed Medical Officer and Public Vaccinator for the Warboys and Wistow District of the St. Ives Union, Hunts. A. Edge, M.B.Lond., M.R.C.S., L.R.C.P., appointed Assistant House Surgeon to the Metropolitan Hospit il, Kingsland Road. F. de Havilland Hall, M.D., F.R.C.P.Lond., appointed Joint Lecturer on Medicine at the Westminster Hospital School of Medicine. F. R. Howard, L.D.S.R.C.S.Eng., appointed Dental Surgeon to the Children's Hospital, Birmingham. R. Johnson, M.B.Lond., B.S., F.R.C.S.Eng., appointed Assistant Surgeon to University College Hospital. W. Kirkpatrick, M.D., B.Ch.Dub., appointed House Surgeon to the Stourbridge Dispensary. Wellington Lake, D.P.H.Camb., M.R.C.S.Eng., appointed Medical Officer of Health to the New Woking Local Board. J. B. Lendrum, M.B., C.M., appointed Assistant House Surgeon to the Huddersfield Infirmary. J. Maclaren M.B. and C.M.Edin., appointed Assistant Medical Officer to the East Riding Asylum, Beverley. G. B. Mower-White, M.B., B.S. Lond., F.R.C.S.Eng., appointed Surgeon to the Out-Patients at the Great Northern Central Hosptal, Holloway Road. W. R. Naylor, M.R.C.S., L.R.C.P., appointed Senior House Surgeon to the Huddersfield Infirmary. J. Priestley, B.A., M.D., D.P.H., appointed Medical Officer of Health for Leicester. L. W. Rolleston, M.B.Durh., M.R.C.S., L.R.C.P., appointed House Surgeon to the Metropolitan Hospital, Kingsland Road. G. Nicholson Stathers, M.R.C.S.Eng, L.R.C.P.Edin., D.P.H. Camb., appointed Medical Officer of Health to the Brackley Rural Sanitary Authority. W. A. Turner, M.D.Edin., M.R.C.P. Lond., appointed Assistant Physician to the West London Hospital, Hammersmith. J. A. S. Wight, M.R.C.S., L.R.C.P., appointed House Physician to the Metropolitan Hospital, Kingsland Road. Mr. F. S. Wood, appointed Assistant Medical Officer to the Workhouse and Infirmary of the parish of Paddington.

### VACANCIES.

#### Resident Medical Officers.

Albany General Hospital, Grahamstown, South Africa. Resident Medical Officer. Salary £200 per annum, increasing yearly by £50 up to £300, with furnished apartments. The selected candidate will have a free first-class passage. Applications to Dr. Macphail, Rowditch, Derby.

Birmingham and Midland Free Hospital for Sick Children, Steelhouse Lane, Birmingham. Resident Medical Officer and Resident Surgical Officer and Extra Acting Physician. Salaries £70, £50, and £60 respectively. The two former will receive board, washing, and attendance. Applications for the first two to the Secretary by December 6th, and for the latter to the Medical Committee by December 6th.

Chelsea Hospital for Women, Fulham Road, S.W. Resident Medical Officer. Salary £80 per annum, with board and residence. Appointment for one year. Applications to the Secretary by December 5th.

Chester General Infirmary. House Surgeon; doubly qualified. Salary £90 per annum, advancing £10 yearly, with residence and maintenance in the house. A sum of £5per annum is granted for taking charge of the library. Applications to the Chairman of the Board of Management by December 4th.

East London Hospital for Sick Children, Glamis Road, Shadwell, E. House Surgeon. No salary. Board and lodging provided. Applications to the Secretary by November 29th.

Kent and Canterbury Hospital. Assistant House Surgeon; unmarried. Salary £50 per annum, with board and lodging. Applications to the Secretary by November 30th.

### SEAMEN'S HOSPITAL SOCIETY, "DREADNOUGHT."

WANTED, HOUSE SURGEON for Branch Hospital, Royal Victoria and Albert Docks, E.

Salary £75 per annum, with Board and Residence.

Candidates must be doubly qualified and registered.

Applications and copies of not more than three testimonials to be sent in on or before TUESDAY, November 28th, to the undersigned, from whom further particulars can be obtained.

By order,

P. MICHELLI Secretary.

Seamen's Hospital Society,

Greenwich, S.E.,

November 14th, 1893 (1,033)

### CHESTER GENERAL INFIRMARY.

### THE HOSPITAL FOR SICK CHILDREN, GREAT ORMOND STREET, W.C.

### HOSPITAL OF ST. PETER, PORT GUERNSEY.

Kimberley Hospital, Kimberley. Senior House Surgeon. Salary £650 per annum, with unfurnished quarters for single men. Applications to H. A. De Beer, Secretary, by December 25th.

National Sanatorium for Consumption and Diseases of the Chest, Bournemouth. Resident Medical Officer and Secretary. Salary £120 per annum, with board and lodging. Applications to the "Chairman of Committee" by December 5th.

New Hospital for Women, 144, Euston Road. Female Resident Medical Officer, and two Female Clinical Assistants for Out-patient Department. Applications to Margaret M. Bagster, Secretary, by November 30th.

Royal Free Hospital, Gray's Inn Road. Senior Resident Medical Officer; doubly qualified. Salary £100 per annum, board and washing. Application to the Secretary by December 11th.

Seamen's Hospital Society "Dreadnought." House Surgeon for Branch Hospital at Royal Victoria and Albert Docks, E.; doubly qualified. Salary £75 per annum, with board and residence. Applications to P. Michelli, Secretary, Seamen's Hospital Society, Greenwich, by November 28th.

Sunderland Infirmary. House Physician; doubly qualified. Salary £80 per annum, rising £10 annually to £100, with board and residence. Applications to the Chairman of the Medical Board by December 4th.

Wolverhampton and Staffordshire General Hospital, Wolverhampton. Resident Assistant. Appointment for six months. Board, lodging, and washing provided. Applications, inscribed "Application for Resident Assistant," to the Chairman of the Medical Committee by November 30th.

### Non-Resident Medical Officers.

Royal Berks Hospital, Reading. Physician. Applications to the Secretary at least ten days before the election on December 12th.

East London Hospital for Sick Children, Glamis Road, Shadwell, E. Two Assistant Physicians; must be F. or M.R.C.P. And Pathologist and Registrar. Honorarium of £40 per annum for the latter. Applications to the Secretary by November 29th.

Guy's Hospital. Demonstrator of Anæsthetics, and two more Anæsthetists. Applications to the Treasurer, Counting House, Guy's Hospital, S.E., by December 1st.

Hospital for Diseases of the Throat, Golden Square, W. Honorary Assistant Physician and Honorary Assistant Surgeon. Applications to the Secretary by November 27th.

Hospital for Sick Children, Great Ormond Street, W.C. Assistant House Surgeon, non-resident. Appointment for six months. Salary, £20. Assistant Physician; must be F. or M.R.C.P.Lond. Applications to the Secretary by November 28th.

Hospital of St. Peter Port, Guernsey. Surgeon. Salary £50. Applications to the President of the Poor-law Board by November 30th.

### PASS LISTS.

### Royal College of Physicians of Edinburgh, Royal College of Surgeons of Edinburgh, and Faculty of Physicians and Surgeons of Glasgow,

The quarterly Examinations in Edinburgh for the Triple Qualification took place in October and November with the following result:—

First Examination.—*Under the entire Examination System.*—Of 34 candidates, the following 18 passed: A. Branson, Rotherham; P. B. Unwin, Wigan; Esther M. Stuart, London; H. O'Donohoe, Queen's County; Margaret G. Todd, Kilrenny; W. Bell, Birkenhead; G. A. I. MacKay, Australia; W. R. Evans, Wales; J. H. E. Trout, Birmingham; Rose G. Rajulu, Madras; C. S. Wilkinson, Shaftesbury; E. Blair, South Shields; J. H. Abrahams, Jamaica; O. Hall, Madras; A. J. Nevett, Shrewsbury; H. T. Wright, Shrewsbury; J. Shand, Morayshire; and G. S. Collett, India.—*Five Years Course.*—Of 8 candidates, the following 5 passed: E. Ryan, County Cork; T. J. Walsh, Kilkenny, R. N. Woodley, County Cork; G. H. A. Taylor, Aryshire; and A. J. Laurie, Allahabad. Three candidates entered for the respective divisions and passed.

Second Examination.—Of 76 candidates, the following 31 passed: W. J. Reid, India; M. Hall, Southport; Margaret G. Todd, Kilrenny; R. W. Devey, Sussex; S. B. Siddall, Liverpool; J. Ingram, County Down; S. O. Hall, India; R. Wolfendale, Tutbury; W. Bell, Birkenhead; C. W. Laver, Melbourne; J. A. Meeke, County Armagh; P. A. Winckler, India; F. A. Routch, Buenos Ayres; J. B. Stewart, County Londonderry; J. M. Murphy, County Kerry; C. G. C. Scudamore, Surrey; A. P. Chapman, London; J. H. E. Trout, Birmingham; J. Johnston, County Derry; R. K. Imeary, Durham; A. Duncan, County Derry; F. Jeeves, Belfast; S. F. Blakely, West Indies; W. J. Collins, Clonmel; G. T. H. De Clive-Lowe, Bangalore; G. Bryce, Whitburn; J. O'Sullivan, County Cork; J. W. Shiels, San Francisco; Annie Gillespie, Newry; H. M. MacLeod Mackenzie, Cumberland; and J. Gallie, Queenstown. Of 24 candidates who entered for the respective divisions 12 passed.

Final Examination.—Of 149 candidates, the following 76 passed and were admitted L.R.C.P.E., L.R.C.S.E., L.F.P., and S.G.: S. Wilson, Hanley; C. A. Foyl, Liverpool; P. Twomey, Drumcollogher; J. R. Whitaker, Lancashire; J. English, Armagh; J. A. Swainson, Harrogate; W. Daunt, Dublin; T. D. S. Shaw, Herts; K. McElfatrick, Derry; A. McAfee, County Antrim; Florence U. O'Sullivan, Killarney; J. J. Gray, Dublin; L. W. Harvey, County Cork; W. M. Sheen, Gravesend; C. G. C. Scudamore, Clapham; F. Isherwood, Darwen; R. S. H. Fuhr, Belfast; J. Ewing, County Antrim; Winifred A. Westlake, Chippenham; G. W. Brabyn, Cornwall; C. Dolman, Devon; H. T. Hollings, Farsley, Leeds; W. M. Keal, Oakham; Sophia Chamney, Dublin; G. G. Roe, Mayo; L. Bostock, Haslington; J. F. W. Ward, India; Alice H. A. Boyle, Dublin; J. I. Twohig, County Cork; W. Candlish, Alnwick; F. A. Hadden, India; Talsat Mansour, Cairo; J. Wallace, County Antrim; H. M. Green, Worthing; A. G. McKenna, Loughrea; A. W. Mason, Glasgow;

L. J. F. Dombren, Mauritius; E. R. Coffey, County Cork; A. P. Chapman, London; D. A. Greaves, Barbados; S. P. Smith, Swindon; J. J. Fanning, Roscrea; M. McGann, County Clare; G. Moore, Newcastle West; W. H. O. Garde, County Cork; A. P. Ross, Windsor; J. H. Collier, Demerara; J. Pirie, Aberdeenshire; W. Lawson, Hull; J. P. Milne, Calcutta; Effield L. Greene, Newport, Monmouthshire; H. F. Ealand, London; W. P. Dunsmore, Devonport; C. Peddie, Dufftown; J. Waddell, Airdrie; M. Donovan, County Cork; S. L. Potter, Oldham; W. Thomas, Llangefni; D. J. Buckley, Mallow; A. H. Brown, London; P. F. James, Glamorganshire; G. H. Lodge, Rotherham; A. F. Hepworth, Dover; A. J. Neill, Melbourne; Susan Campbell, Greenock; J. M. Sloan, Huron County; P. D. Fethers, Victoria; C. E. G. Bateman, Norwich; P. R. Cairns, Galashiels; H. L. McCulloch, Victoria; A. Robertson, Dunkeld; R. G. Jayetileke, Ceylon; W. Yeates, County Down; J. Tait, Moffat; L. Bowman, New Zealand; and P. W. Griffiths, Merthyr Tydfil. Of 28 candidates who entered for the respective divisions 19 passed.

The following candidates, having passed the requisite examinations held in October, received the Diploma in Public Health of the three corporations: A. G. Robertson, M.B., C.M., Edinburgh; D. G. Braidwood, M.B., C.M., Edinburgh; and W. A. Winter, M.B., B.Ch., Dublin.

### Royal College of Surgeons of Edinburgh.

The following candidates, having passed the requisite examination on July 21st, 1893, were elected Fellows of the College at a meeting held on October 18th, 1893: W. A. Betts, L.R.C.S.E., Royal Southern Hospital, Liverpool; F. Winson Ramsey, M.D., L.R.C.S.E., Jesmond Dene, Bournemouth; H. B. Rygate, M.R.C.S.Eng., 61, Loraine Place, Newcastle-on-Tyne; T. H. Littlejohn, M.B., C.M.Edin., 24, Royal Circus, Edinburgh; C. A. Starrock, M.B., C.M.Edin., 15, Regent Terrace, Edinburgh; M. O. Ramsay, L.R.C.S.E., Closeburn, Thornhill, Dumfries-shire; and G. E. Clemons, M.B., C.M.Edin., 54, Marchmont Road, Edinburgh. Surgeon-Colonel C. McDonagh Cuffe, C.B., L.R.C.S.E., Kembella House, Antrim Road, Belfast, was elected a Fellow of the College without examination.

The following candidates, having passed the requisite examination, were admitted Licentiates of the College in October, 1893: T. B. Richardson, Canada; and A. H. Nichol, Canada.

### THE EDINBURGH TRIPLE QUALIFICATION.

**Papers Recently Set.**

FINAL EXAMINATION.

QUESTIONS IN MEDICINE. (Four Questions, of which Three only are to be answered.)

1. Describe a typical attack of Tertian Ague.
2. What are the causes of Pulmonary (Vesicular) Emphysema? What symptoms and physical signs would you expect to be present in a well marked case of Pulmonary (large-lunged) Emphysema?
3. Describe the symptoms characteristic of Chlorosis. How would you distinguish a case of Chlorosis from a case of Pernicious Anæmia?
4. Describe the chief clinical features of Pseudo-Hypertrophic Paralysis.

QUESTIONS IN MEDICAL JURISPRUDENCE AND HYGIENE. (Only Three Questions to be answered, including Nos. 3 and 4.)

1. How might you determine from the dead body of an infant whether or not it had been living at its birth; and, if born alive, how long it had lived?
2. A man, apparently between forty and fifty years of age, has been found unconscious in the street, with contracted pupils; no further history is available. To what may his condition be due? Detail the points you would investigate before arriving at a diagnosis, and how you would treat him.
3. What are the symptoms and treatment of poisoning by Carbolic Acid? By what chemical test would you verify its presence?
4. What is the composition of the atmosphere of a city and that of the open country? State the effects produced on man and animals by impure air, and how they are manifested.

QUESTIONS IN SURGERY. (Four Questions, of which Three only are to be answered.)

1. In what class of cases is distal ligature of an artery for Aneurism suitable, and what are the risks?
2. Describe the signs and symptoms of Abscess of the Antrum, and the treatment.
3. Describe an Aneurismal Varix and a Varicose Aneurism.
4. How is a Fæcal Fistula in the inguinal region produced, and what are the best methods of treatment?

SURGICAL ANATOMY. (Both of which are to be answered.)

1. What Muscles are attached externally and internally to the Ramus and Body of the Inferior Maxilla?

3. Describe the Vertebral articulations in the dorsal region, and name the ligaments connecting them.

QUESTIONS IN MIDWIFERY AND GYNÆCOLOGY. (Only Three Questions to be answered, of which the Fourth must be one.)

1. Describe the methods for determining the length of the Antero-posterior diameter of the Pelvis at the brim.
2. Describe the mechanism of Labour in the Third Cranial position.
3. Describe the symptoms of Hour-glass Constriction of Uterus, and the treatment.
4. What are the principal means of diagnosis of Epithelioma of the Os and Cervix Uteri?

